# Introduction from Guest Editors to Special Issue “Multi-Metallic Systems: From Strong Cooperative Bonds to Weak M-M Interactions”

**DOI:** 10.3390/ijms231910998

**Published:** 2022-09-20

**Authors:** Mauro Fianchini, Oleg V. Mikhailov

**Affiliations:** 1 MagnetoCat SL Calle General Polavieja 9, 3Iz, 03012 Alicante, Spain; 2 Department of Analytical Chemistry, Certification and Quality Management, Kazan National Research Technological University, K. Marx Street 68, 420015 Kazan, Russia

As is known, the chemical elements called metals make up the majority of all the chemical elements included in the periodic table of D.I. Mendeleev. Metal elements (hereinafter referred to as M) include all *s*-, *d*- and *f*-elements, and, also, some of the *p*-elements, namely Al, Ga, In, Tl, that are in the III (XIV) group, Sn, Pb (in the IV (XV) group) and Bi (in the V (XVI) group). These elements form a wide variety of chemical compounds; among them, a very important place is occupied by those substances in which metal atoms are chemically bonded to each other due to so-called “socialized” electrons. Two main types of chemical bonds formed by atoms of metal elements with each other occur: **metallic**, which is a multicenter bond and formed due to electrostatic interactions between metal ions and free electrons occupying voids between the nodes of the crystal structure (“lattice”), and **covalent**, formed within the framework of the interaction between at least two nuclides and two “socialized” electrons (which, with a sufficiently significant difference in the electronegativity of the atoms of the elements, can be transformed into a quasi-ionic bond). Three categories of substances in which there are such chemical bonds, can be emphasized:

- compounds with a **purely metallic bond** (simple substances formed by metal elements, their alloys, and, also, certain intermetallic compounds);
- compounds with a **mixed types of bonds** (metallic, covalent and/or ionic) between metal atoms (intermetallic compounds);
- compounds with purely covalent bonds formed by at least two atoms of metal elements (bi- and polynuclear compounds containing various chemical bonds and at least one covalent metal–metal bond—so-called metal clusters or simply clusters).

Different types of M-M bonds in chemical substances also imply different degrees of interaction between them—from strong cooperative bonds, which take place in the case of the formation of a metal bond, to very weak ones, which manifest themselves in the framework of intermolecular interactions (e.g., the molecular structure of mercury presents diatomic Hg_2_ clusters in the nodes). In this regard, it is important to distinguish between the concepts of “metallic bond” and “metal-metal bond”, the first of which refers only to a multicenter bond in a metal crystal structure, while the second, as a rule, to a covalent bond.

Compounds of the first of the above three categories have found their application in anthropogenic activities since ancient times; our life is unthinkable without them at the present time (although a considerable part of the “niche” that they have been occupying for a long time has now been occupied by other materials). These include, without exception, all homonuclear compounds formed by atoms of *s*-, *d*- and *f*-elements, in a solid crystalline or amorphous state, as well as in their melts when at relatively low temperatures. The key components of the chemical bond in these compounds are electrons that move freely in the space between lattice sites (the so-called “electron gas”) and ions formed as a result of the loss of such electrons by atoms of certain (but, by no means, all) chemical elements. Only those chemical elements that have a relatively low electronegativity and quite easily donate their outer (valence) electrons when in contact with each other, in fact, are capable of forming substances with this type of bond. As a result, metal cations appear, which form the nodes of the crystal lattice and “socialized” electrons, that move easily and freely throughout the entire volume of the substance. According to Coulomb’s law, electrostatic interactions arise between ions and free electrons; these interactions, namely, are the root cause of the appearance of a metallic bond. On the other hand, when a large number of atoms (*N*) with relatively low electronegativity approach each other within the framework of the crystal lattice, the overlap of electron clouds of neighboring atoms leads to the splitting of the energy levels of valence electrons into *N* sublevels. The energy gaps between split levels are so small that their totality can be considered a practically continuous band of energy levels with finite width. This situation becomes possible because the number of atomic orbitals (AO) participating in the formation of a such a bond far exceeds the number of electrons participating in its formation. As it is easy to see, each atom here participates, with the same number of valence electrons, in the formation of a larger framework of M-M bonds than in the case of pure covalent bonds. Thus, a metallic bond is one of the varieties of cooperative interaction and, therefore, can take place only if there is a sufficiently large (at least several hundred) number of atoms of the corresponding chemical element. This aspect distinguishes it from all other types of chemical bonds, which in principle can be formed by the participation of only two atoms (ionic, covalent, hydrogen, van der Waals). The metallic bond is delocalized, non-directional and unsaturated and leads to the common properties as a characteristic (“metallic”) luster (due to the pronounced ability to reflect light), high melting points, thermal and electrical conductivity, malleability and plasticity.

Compounds of the second category are represented by a very significant number of so-called intermetallic compounds (intermetallics), for which the metallic bond is to some extent combined with another type of chemical bond (i.e., the bond between atoms in intermetallics is transitional from metallic to covalent or even quasi-ionic). The formation of a covalent bond takes place in cases where the intermetallic composition includes metal atoms with close electronegativities: for example, compounds such as Pb_2_Au, Ni_3_Nb, and Cu_6_Sn_5_ are intermetallic. A quasi-ionic bond is realized with a significant difference in the electronegativities of the metals forming it. In this case, as a guiding rule, one of these metals is some *s*-element: MgAg, AlAu_4_, and Na_4_Sn belong to this category. There are also intermetallic compounds where the presence of a predominantly ionic bond between atoms is postulated: CsAu, in particular, belongs to this category. The appearance of any of such two types of bonds in the structure of these compounds is accompanied by weakened “metallicity”, decreased plasticity (ductility) and increased electrical resistance (moreover, some of the intermetallic compounds even turn out to be semiconductors). In this regard, many intermetallic compounds are less ductile than the parent metals forming them and impart increased brittleness to the structures of alloys where they are included. 

The following fact is also noteworthy: most metals are known to be gray to white in color with the exceptions of copper and gold, which have red and yellow colors, respectively. Intermetallics, however, have a much greater variety of colors, and typically their color is not the result of a simple combination of the colors of the forming metals. So, Al_2_Au is violet, RbAu_2_ dark green, In_2_Pt pinkish yellow and InPd purple-red (in the last two cases, the metals that form these intermetallic compounds—indium, platinum and palladium—do not have any visually noticeable color). A special group of intermetallic compounds is formed by the so-called nano-intermetallic compounds having a specific molecular structure and existing as separate molecules; the chemical bonds between the atoms of such compounds are purely covalent. Compounds of this category have also found rather significant practical applications in various branches of anthropogenic activities.

The third category includes chemicals that have received the collective name of “clusters”. This term was introduced into the scientific community by F.A. Cotton in 1964. According to him, a cluster is a chemical compound containing metal atoms that are wholly or largely linked by covalent bonds to each other (for extension, the composition of this compound may also contain non-metal atoms that are part of any structural groups associated with metal atoms). Initially, this term referred almost exclusively to coordination and organometallic compounds of *p*-, *d*-, and *f*-elements. Over time, however, more and more new substances have been included in it, such as compounds containing only atoms of non-metal elements (e.g., fullerenes and boranes). At the present time, the concept of “cluster” includes all chemical compounds that are intermediate between a molecule and a bulk solid with the most diverse stoichiometric composition and geometric structure. In modern chemistry, nanoparticles of an ordered structure, having a given packing of atoms and a regular geometric shape, have also been included among them. We will be guided by the classical definition of the term “metal cluster”, because the items included in this group are still of great interest after more than half a century and this interest is only increasing year by year.

In the first approximation, two types of items fall under this definition and they can be distinguished into supported metal clusters (i.e., ligand clusters), in which the carcass of metal atoms is bound to certain organic and/or inorganic compounds, usually called ligands (e.g., triosmium(0) decacarbonyl dibromide Os_3_(μ_2_-Br)_2_(CO)_10_, in which carbon monoxide CO molecules and Br^–^ anions act as a ligands), and unsupported metal clusters (i.e., non-ligand clusters), in which such “binding” is absent (e.g., Nb_2_Pd_3_Se_8_). Metal clusters of the second of these varieties, in turn, are also divided into two groups: metallic, containing only atoms of metal elements, and non-metal-metallic, containing, along with atoms of metal elements, also atoms of non-metal elements (sulfur, carbon, phosphorus, etc.). The diversity within the metal clusters group is quite significant; among them there are even real giants where the number of metal atoms; hence, the number of M-M bonds, is many hundreds and even thousands: a good example is [Pd_561_(phen)_60_(OAc)_180_], where phen is 1,10-phenanthroline and OAc is an acetate anion.

Increased attention to chemical compounds presenting various and diverse metal-metal interactions makes the problem of predicting their physicochemical characteristics their useful properties and, consequently their most suitable applications very relevant. One of the most common problems associated with each of the above categories of metal-containing compounds is an adequate description of the nature of metal–metal (M-M) bonds, which is possible only within the framework of quantum mechanics via quantum chemical modeling. Likewise, the choice of computational methods able to correctly describe and reproduce such metal–metal interactions and their physico-chemical properties is far from trivial.

Density Functional Theory (DFT) is currently the most applied computational method, due to its obvious advantages in terms of computational costs and accuracy, to covalent M-M bonds of various polarization, though multiconfigurational methods, such as CASSCF and CASPT2, should be more conceptually appropriate, albeit computationally more taxing and challenging. Highly correlated post-HF methods (e.g., MP2 or even CCSD(T)) are recommended to reproduce “metallophilic” interactions (e.g., weak interactions between *d*^10^ or *d*^8^ configurations comparable in strength with H-bonds), though DFT, complemented by long-range and empirical dispersion corrections, may lead to favorable accuracy/cost ratios for large systems containing M–M bonds (dispersion-corrected semiempirical and tight binding DFT methods promise to extend reasonable accuracy even to far larger systems). The description of metallic bonds in solids used to rely on LDA DFT and has been improved over the years via GGA and meta-GGA approaches (hybrid functionals on periodic systems are still quite computationally demanding) to make more accurate characterizations. Open-shell magnetic configurations still remain very challenging for computation and typically rely on spin-polarized GGA wavefunctions improved via the introductions of corrections such as the Hubbard term (i.e., DFT + U).

Post-wavefunction NBO, QTAIM, ELF, DORI, LOL, NCI, and RDG analyses (among others) are typically employed to characterize the strength and the nature of M-M interactions. Despite all this, theoretical works devoted to quantum chemical calculations of M-M bonds and interactions by DFT methods (or, sparingly, more advanced and expensive methods), are still sparse in the literature, and, in any case, there are much fewer of theoretical publications than works devoted only to the synthesis and study of the physics-chemistry of these compounds. In this regard, as it seems to the authors of the given editorial, the computational challenges of studying M-M interactions are no less than those of synthesizing and characterizing novel structures with optimal designs and properties to satisfy the postulated goals and both of them need to be addressed.

In view of the foregoing, this Special Issue of the *International Journal of Molecular Science* is intended as a multidisciplinary and interdisciplinary platform on M-M bonds and interactions bringing together various branches of science, primarily coordination, organometallic, supramolecular chemistry, theoretical and computational chemistry and solid-state chemistry. We would like to hope that the idea of its creation will find wide support from researchers in several fields (from theory to applications) and will contribute to the emergence of new ideas, such as structural and catalytic innovations, evidence of cooperative behavior, innovative theoretical approaches and/or modern methods of computer analysis and modeling, as well as the demonstration of synergetic approaches between theory and experiment in this scientific direction.

With this Special Issue of the *International Journal of Molecular Science,* we also aim to fill, to a certain extent, the still existing gap in the theoretical and quantum chemistry of chemical compounds bearing M-M interactions.

